# The Book World of Medicine and Science

**Published:** 1906-12-08

**Authors:** 


### The Book World of Medicine and Science.

Practical Prescribing and Dispensing for Medical Students. By William Kirkby, sometime Lecturer in Pharmacognosy in the Owens College, Manchester. Second edition. (Manchester: at the University Press.)

This practical little work is designed to rectify the complaint that medical students cannot write prescriptions, and to furnish a means of making a practical acquaintance with the various forms in which medicines are administered, and of learning how to avoid prescribing incompatible drugs. It is a practical work and well suited to fulfil the purpose for which it has been designed. It contains also some very useful dispensing exercises, and should find a place in the dispensaries attached to most hospitals, where it will be found most useful as a work of reference should difficulties arise.

Minor and Operative Surgery, including Bandaging. By Henry R. Wharton, M.D., Professor of Clinical Surgery in the Women's Medical College of Pennsylvania; Surgeon to the Presbyterian Hospital and the Children's Hospital; Consulting Surgeon to St. Christopher's Hospital, the Bryn Mawr Hospital, and Girard College; Fellow of the American Surgical Association. Sixth edition, enlarged and thoroughly revised, with 532 illustrations. (London: Rebman, Ltd., 129 Shaftesbury Avenue, W.C.).

This work is thoroughly up to date, profusely illustrated, and simply and carefully written. It deals with all modern aids to surgery, including electrolysis, the galvano-cautery, the use of the cystoscope, the urethrascope, and skiagraphy. The chapter on anæsthetics is well up to date, and a number of operations frequently required in practice are more particularly dealt with. These include tracheotomy, intubation of the larynx, operations for appendicitis, strangulated hernia, and so forth. That it has served its purpose thoroughly and well is indicated by the call for a sixth edition. The illustrations are exquisitely executed, and each conveys its lesson in a graphic form. They are culled from the best works on surgery in Europe, notably from Bryant, Druitt, Esmarch, Smith, Holmes, Liston, Fergusson, and Treves.

